# Trends in statin prescription among osteoporosis patients: A retrospective cohort study using UK primary care data

**DOI:** 10.1371/journal.pgph.0005656

**Published:** 2025-12-29

**Authors:** Ingrid Mei-Fung Wong, Philip Chun-Ming Au, Noel Cassandra Yue, Jonathan Ka-Long Mak, Xiaowen Zhang, Chor-Wing Sing, Kathryn Choon-Beng Tan, Ching-Lung Cheung, Gerard Anderson

**Affiliations:** 1 Department of Health Policy and Management, Johns Hopkins Bloomberg School of Public Health, Baltimore, Maryland, United States of America; 2 Department of Pharmacology and Pharmacy, Li Ka Shing Faculty of Medicine, The University of Hong Kong, Pokfulam, Hong Kong; 3 Department of Medicine, Queen Mary Hospital, Pokfulam, Hong Kong; 4 Hinda and Arthur Marcus Institute for Aging Research, Hebrew SeniorLife, Boston, Massachusetts, United States of America; 5 Department of International Health, Johns Hopkins Bloomberg School of Public Health, Baltimore, Maryland, United States of America; 6 Johns Hopkins University School of Medicine, Baltimore, Maryland, United States of America; The Population Council, UNITED STATES OF AMERICA

## Abstract

Statin prescribing patterns in osteoporosis patients remain uncharacterized despite cardiovascular disease being a leading cause of mortality in this population. This study examined temporal trends and demographic variations in statin prescription rates among osteoporosis patients in the UK. This research is a retrospective cohort study utilizing the IQVIA Medical Research Data (IMRD) spanning 2005–2019. Patients’ diagnosis of osteoporosis and corresponding statin prescription data were extracted. Prevalence and incidence of statin prescription were calculated, followed by age-stratified and gender-stratified trends analysis using joinpoint regression to calculate the average percent change (APC), annual average percent change (AAPC), and identify significant temporal shifts. Among 157,483 identified osteoporosis patients, statin prescribing trends demonstrated significant reductions in both prescription prevalence (AAPC: -1.03%, p = 0.005) and incidence rate (AAPC:-1.82%, p < 0.001). Gender-based analysis revealed higher prescription rates and more pronounced declines in male patients (prevalence AAPC:2.7% vs female -0.8%), resulting in prescription rate convergence by study end. Age stratified findings showed the 70–79 years age group maintained the highest initial prevalence but experienced the steepest declines. A notable breakpoint occurred in 2018 within the 50–59 age cohort, which may be a result of stricter ACC/AHA cholesterol guidelines requiring enhanced risk-based criteria for primary and secondary prevention. The prescription peaks in 2006 and 2014 likely coincided with NICE guideline modifications. Despite these guideline expansions encouraging broader statin use, sensitivity analysis of cumulative defined daily dose revealed persistently high discontinuation rates in osteoporosis patients. These patterns indicate substantial gaps in cardiovascular protection for this vulnerable population. Declining statin prescription trends among osteoporosis patients, particularly among elderly males, appear to reflect an evolution in clinical practices toward risk-benefit assessment. These findings highlight the need for treatment guideline advancement that addresses both appropriate statin initiation and strategies to improve long-term adherence in patients with multimorbidity.

## Introduction

Osteoporosis is a significant public health challenge globally [1,2]. It is characterized by deterioration of bone mineral density and microarchitecture that substantially increases fracture susceptibility [3]. Cardiovascular disease (CVD) and osteoporosis frequently coexist in older adults, creating a complex therapeutic landscape requiring careful clinical decision-making [4,5]. Epidemiological studies demonstrated that individuals with osteoporosis exhibit significantly higher cardiovascular risk profiles compared to age-matched controls, who are individuals of similar age without osteoporosis. Following the major osteoporotic fracture, the risk of cardiovascular events to increases 3–4 fold [5,6]. Recent studies further support this association, showing that hip fracture is temporally linked to multiple cardiovascular events with hazard ratios ranging from 1.97 to 4.06 [7]. This relationship may be mechanistically and partly explained by shared pathophysiological pathways, such as deregulation of Wnt/β-catenin signalling, which have been reported to reduce bone density and may contribute to the heightened cardiovascular risk observed in osteoporotic patients [5,8–10]. Consequently, the interconnected nature of osteoporosis and cardiovascular disease creates a complex therapeutic landscape that necessitates careful clinical decision-making when addressing cardiovascular risk reduction in patients with osteoporosis.

The management of osteoporosis typically focuses on bone-specific interventions, such as bisphosphonates, monoclonal antibodies targeting receptor activator of NFκB ligand (RANKL), selective oestrogen receptor modulators (SERMs), and parathyroid hormone (PTH) analogues [2,11]. However, given the established link between cardiovascular disease and osteoporosis, there is growing interest in therapeutic approaches that could address both conditions simultaneously. The latest American Heart Association (AHA) guidelines recommend statin therapy as first-line treatment for primary prevention of atherosclerotic cardiovascular disease in patients with elevated low-density lipoprotein cholesterol levels (≥190 mg/dL) and those with diabetes mellitus aged 40–75 years [12]. Beyond their established cardiovascular benefits, emerging evidence suggests that statins possess pleiotropic effects that may benefit bone metabolism through enhanced osteoblast differentiation, inhibited osteoclast activity, and anti-inflammatory properties [13–15]. Large database studies support this hypothesis by demonstrating significant fracture risk reduction with statin use [16–18], suggesting dual cardiovascular and skeletal benefits from statin therapy. The use of statins in UK primary care has grown substantially over time, with older patients experiencing the most notable increase in prescription rates [19,20]. Despite documented increases in statin adoption following guideline revisions [20–24], the temporal trends in statin prescribing within osteoporosis patients remain unstudied.

This study aimed to characterize temporal trends in statin prescription prevalence and incidence rates among UK osteoporosis patients from 2005 to 2019; and to understand how policy changes influenced prescribing behaviours and statin utilization patterns in patients. This 15-year study period captured multiple transformative changes in UK statin prescribing practices.

## Methods

### Ethics statement

This study was reviewed and approved by the IQVIA Scientific Review Committee (approval number: 23SRC031, approval granted on 31 January 2024). This research involved secondary analysis of retrospective, de-identified electronic health records from the IQVIA Medical Research Data (IMRD) database; and the authors had no access to information that could identify individual participants at any stage.

### Data source

This study utilized de-identified primary care data from the IQVIA Medical Research Data (IMRD), a Cegedim Database, containing longitudinal electronic health records from over 20 million UK patients, representing approximately 6% of the population [25,26]. In the UK system, 98% of residents are registered with general practitioners who record comprehensive patient information including diagnoses, prescriptions, and laboratory results using the Read code classification system during our study period [27,28]. IMRD has been extensively validated for pharmacoepidemiologic research and demonstrated representativeness of the UK population in demographics and disease prevalence, making it well-suited for multimorbidity epidemiological studies [29–31].

### Study population

This study examined patients aged 50 years or older with newly diagnosed osteoporosis between January 1, 2005, and December 31, 2019 in UK prior the COVID-19 pandemic. The age threshold of 50 years was chosen because osteoporotic fracture incidence increases substantially after this age [32]. The study population included only active patients who remained registered with UK primary care practices covered by the IMRD as of the data collection date. The study population was systematically identified following IMRD data manual recommendations, with diagnosed osteoporosis patients captured using comprehensive Read codes detailed in S1 Text. Among osteoporosis patients, statin prescribing patterns were assessed using Read codes S2 Text. to categorize patients as either new statin users or prevalent statin users.

### Statin prescription definition

Statin exposure was defined as receiving at least one prescription for any commonly prescribed statins, including atorvastatin, fluvastatin, pravastatin, rosuvastatin, or simvastatin, in the UK primary care setting during the study period. The first statin prescription date was determined for each patient based on their earliest recorded statin prescription during the study period since January 1 2005, such that each patient had at least one year screening period.

### Statistical analysis

Incidence rate was calculated as the number of statin-naive patients who initiated statin therapy for the first time within one year from their osteoporosis diagnosis, divided by the total person-years of observation for the at-risk population. The at-risk population included only patients with no statin exposure in the 6 months preceding their osteoporosis diagnosis. Patients contributed person-time from their osteoporosis diagnosis date until they either initiated statin therapy (at which point they were censored), died, transferred out of the practice, reached the end of the one-year follow-up period, or reached the end of the study period, whichever occurred first.

Prevalence was calculated as the number of patients who had any statin prescription exposure either within the 6 months preceding their osteoporosis diagnosis or at any time during the calendar year of their diagnosis, divided by the total number of patients diagnosed with osteoporosis in each respective calendar year.

Joinpoint regression analysis identified significant temporal trend changes in statin for both prevalence and incidence rate (2005–2019) using piecewise linear regression with log-linear transformation. Model selection employed Bayesian Information Criterion with complexity penalty, comparing 0–3 joinpoints with minimum 2-year intervals. Breakpoint significance was assessed through permutation tests, with Average Annual Percent Change (AAPC) and Average Percent Change (APC). Fitted trend lines represent the best-fitting segmented regression model, with 95% confidence intervals shown as shaded areas.

Sensitivity analysis using varying cumulative defined daily dose (cDDD) was conducted to assess the robustness of statin utilization trends of prevalent users and incident users. Six cDDD thresholds were used to capture varying degrees of treatment exposure. The lower thresholds, including ≥1 DDD (main analysis), ≥ 30 DDD (~1 month), ≥ 90 DDD (~3 months) and ≥180 DDD (~6 months) identify any exposure attempt including brief trials; while higher thresholds including ≥365 DDD (~1 year) and ≥730 DDD (~2 years) represent sustained therapeutic period. For each cDDD threshold, the proportion of exposed individuals was calculated and expressed as rates per 100 person-years to account for varying population sizes of various level of cumulative statin exposure.

All analyses were performed using R version 4.1.0 (R Foundation for Statistical Computing, Vienna, Austria).

## Results

### Demographic distribution of osteoporosis patients aged 50 or above

**Fig 1:**
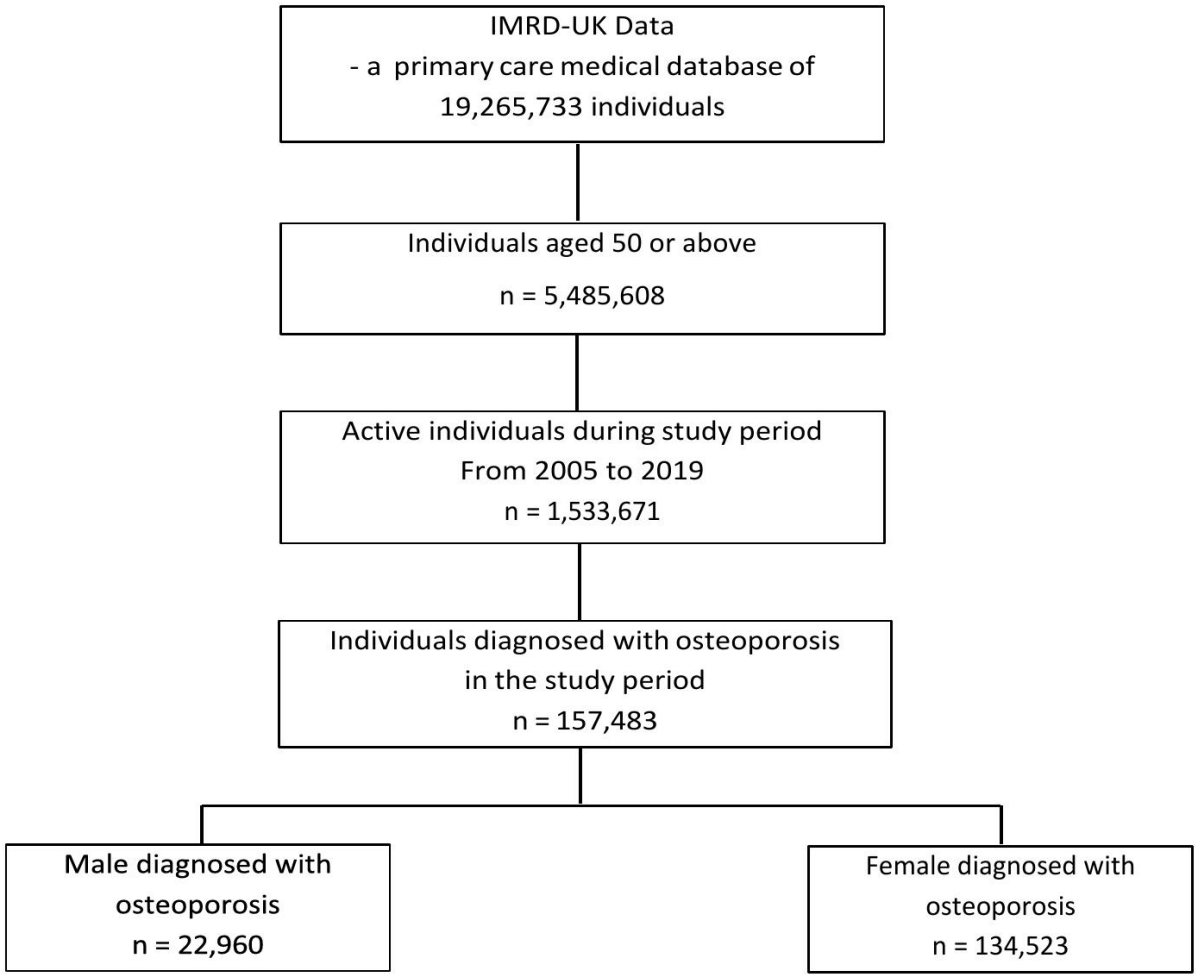
Study patient flow.

Among the 5,485,608 patients aged 50 years or older, 1,533,671 were active patients during the study period. Of these active patients, 157,483 individuals were diagnosed with osteoporosis between 2005 and 2019. Stratification by sex within this osteoporosis cohort revealed a pronounced gender disparity, with 22,960 males (14.58%) and 134,523 females (85.42%) meeting the inclusion criteria. The flow of patient selection was summarized in Fig 1, and Table 1 showed the age distribution of osteoporosis diagnosis. The results revealed the osteoporosis patients aged 70–79 years account for 31.71% (n = 49,932) and patients aged 80 or above account for 31.04% (n = 48,885). Patients aged 60–69 years represented 39,199 cases (24.89%), while those in the 50–59 years age group accounted for the smallest proportion with 19,466 diagnoses (12.36%).

**Table 1:** Demographics of osteoporosis study cohort.

	Male		Female		Total number of patients
**Osteoporosis cohort**	22,960	(−14.58%)	134,523	(−85.42%)	157,483
**Age distribution**					
**50–59**	3,141	(−16.14%)	16,325	(−83.86%)	19,466
**60–69**	5,471	(−13.96%)	33,728	(−86.04%)	39,199
**70–79**	7,238	(−14.5%)	42,694	(−85.5%)	49,932
**80+**	7,109	(−14.54%)	41,776	(−85.46%)	48,885

### Temporal trends of prevalence and incidence in statin prescription patterns among osteoporosis patients, 2005–2019

The 15-year temporal trends in statin prescription prevalence and incidence rate among diagnosed osteoporosis patients from 2005 to 2019 (S1 Table), the average annual percentage change (AAPC) and Average Percent Change (APC) of the trend in subgroups were summarized in Table 2.

**Table 2:** Temporal trends in sex- and age- stratified statin prescription prevalence and incidence rates analyzed using joinpoint regression.

		2005	2019	Year	AAPC	(95% CI)	p value	Year	APC (95% CI)
Prevalence	Overall	33.76	31.15	2005–2019	−1.03*	(−1.68, −0.38)	0.005		
	Male	49.56	35.79	2005–2019	−2.77*	(−4.64, −0.86)	0.008		
	Female	31.83	30.23	2005–2019	−0.80	(−1.68, 0.09)	0.075		
	50-59 years	26.29	15.58	2005–2019	−0.92	(−2.90, 1.10)	0.338	2005–2018	0.44 (−0.70, 1.58)
								2018–2019	−38.81^a^ (NaN, NaN)
	60-69 years	34.31	34.83	2005–2019	−0.32	(−1.44, 0.80)	0.546		
	70-79 years	36.40	32.98	2005–2019	−1.66*	(−3.12, −0.18)	0.031		
	80 + years	33.47	33.47	2005–2019	−0.96	(−2.16, 0.25)	0.111		
Incidence	Overall	22.71	17.54	2005–2019	−1.82*	(−2.67, −0.96)	<0.001		
	Male	29.32	14.85	2005–2019	−3.41*	(−5.90, −0.86)	0.013		
	Female	21.91	18.08	2005–2019	−1.59*	(−2.51, −0.67)	0.003		
	50-59 years	14.08	12.50	2005–2019	−0.41	(−2.52, 1.75)	0.688		
	60-69 years	24.28	19.37	2005–2019	−1.13	(−3.01, 0.78)	0.222		
	70-79 years	23.80	18.05	2005–2019	−2.77*	(−4.19, −1.32)	0.001		
	80 + years	23.94	17.80	2005–2019	−1.87*	(−3.54, −0.18)	0.033		

^a^ Confidence interval is not estimated due to insufficient degrees of freedom in the statistical model.

**Fig 2:**
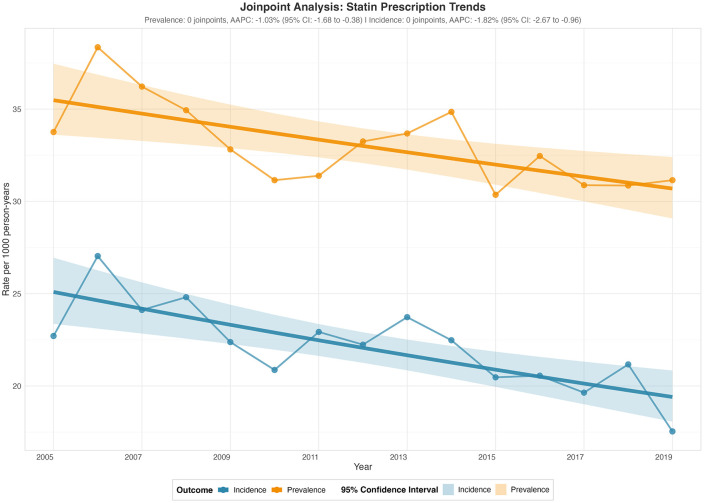
Jointpoint analysis on statin prescription trends.

Statin prescription prevalence demonstrated a modest but statistically significant declining trend over the study period (AAPC: -1.03%, 95% CI: -1.68 to -0.38; p = 0.005) (Fig 2) (Table 2). Statin prescription prevalence peaked in 2006 and subsequently declined. The incidence showed a steeper and statistically significant downward trajectory compared to prevalence (AAPC: -1.82%, 95% CI: -2.67 to -0.96; p < 0.001)(Table 2). Incidence rates reached their maximum in 2006 at 27.04 per 1,000 persons before demonstrating a sustained downward trend.

### Gender-stratified analysis of statin prescription

#### Prevalence.

**Fig 3:**
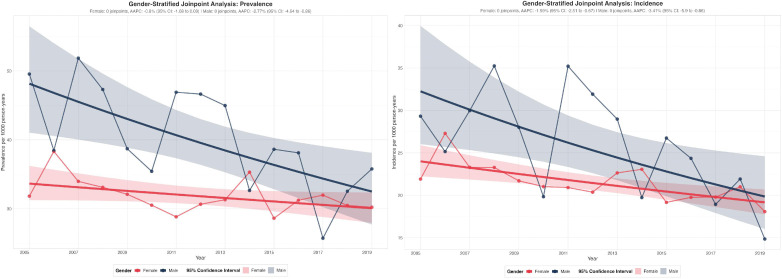
Statin prescription prevalence stratified by gender.

Statin prescription prevalence among female osteoporosis patients remained stable without significant temporal trends (AAPC: -0.8%, 95% CI: -1.68 to 0.09, p = 0.075), from 28 to 38 per 1,000 persons (Fig 3, S2 Table). Conversely, male patients exhibited significant declining trends (AAPC: -2.77%, 95% CI: -4.64 to -0.86, p = 0.008), decreasing from 49.56 per 1,000 persons in 2005 to 25.73 per 1,000 persons in 2017. Males consistently demonstrated higher prescription prevalence than females throughout most of the study period, with the gender gap most pronounced during 2005–2013 (15–20 per 1,000 persons difference). However, the gender gap narrowed over time as the gender difference reduced to approximately 5 per 1,000 persons by 2019.

#### Incidence.

Statin prescription incidence demonstrated significant declining trends in both genders (Fig 3), with females showing modest declines (AAPC: -1.59%, 95% CI: -2.51 to -0.67, p = 0.003) from 21.91per 1,000 person-years in 2005 to 18.08 per 1,000 person-years in 2019. Males exhibited more pronounced declines (AAPC: -3.41%, 95% CI: -5.90 to -0.86, p = 0.013), decreasing from 29.32 per 1,000 person-years in 2005 to 14.85 per 1,000 person-years in 2019. Males consistently demonstrated higher incidence rates than females during 2005–2015, with differences ranging from 5-15 per 1,000 person-years. However, gender convergence occurred by 2017–2019, with both groups showing similar rates around 15–20 per 1,000 person-years. The steeper decline in male incidence rates ultimately resulted in gender parity by the end of the observation period.

### Age-stratified analysis of statin prescription

#### Prevalence.

**Fig 4:**
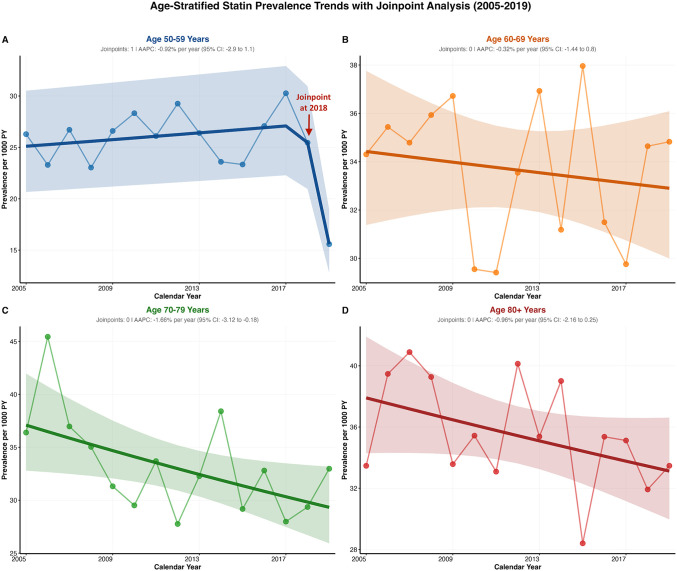
Age-stratified statin prevalence trend with Jointpoint analysis.

Age-stratified analysis revealed distinct patterns in statin prescription prevalence (Table 2 and S3 Table). The 50–59 years group showed a biphasic pattern with a joinpoint in 2018 (AAPC: -0.92%, 95% CI: -2.9 to 1.1, p = 0.338), remaining stable until 2018 then declining dramatically from 2018-2019. The confidence interval for breakpoint was not estimable due to insufficient degree of freedom. The 60–69 years group maintained stable rates throughout the study period (AAPC: -0.33%, 95% CI: -1.44 to 0.8; p = 0.546), see Fig 4. The 70–79 years group demonstrated the steepest significant decline (AAPC: -1.66%, 95% CI: -3.12 to -0.18, p = 0.031) from the highest initial prevalence, while the 80 + years group also showed non-significant declining trends (AAPC: -0.96%, 95% CI: -2.16 to 0.25, p = 0.111). Notable convergence occurred by 2017, with all age groups reaching similar prevalence around 30.0 per 1,000 persons despite markedly different starting points and trajectories.

#### Incidence.

**Fig 5:**
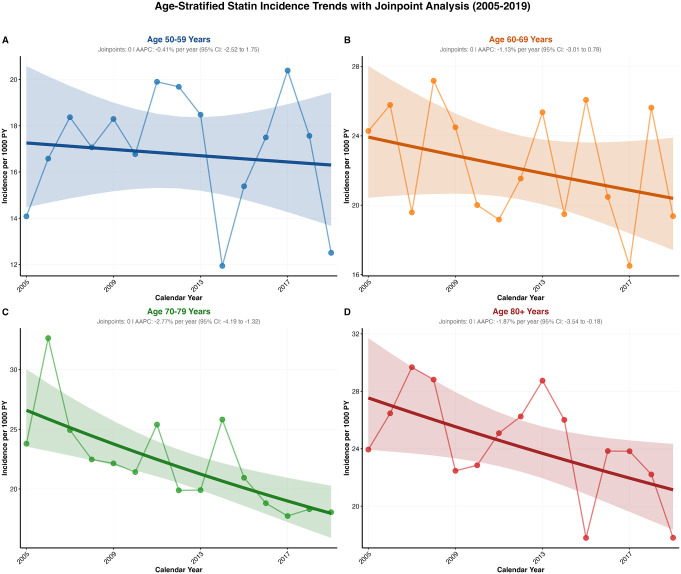
Age-stratified statin incidence trend with Jointpoint analysis.

Age-stratified statin prescription incidence revealed distinct patterns across four age groups (S4 Table). The 50–59 years group showed non-significant trends (AAPC: -0.41%, 95% CI: -2.52 to 1.75, p = 0.688) with substantial variability, while the 60–69 years group exhibited modest non-significant declines (AAPC: -1.13%, 95% CI: -3.01 to 0.78, p = 0.222) but maintained consistently higher incidence rates throughout the study period. The 70–79 years group demonstrated the steepest significant decline (AAPC: -2.77%, 95% CI: -4.19 to -1.32, p = 0.001), decreasing from 23.8 to 18.05 per 1,000 person-years between 2005–2019. The 80 + years group also showed significant declining trends (AAPC: -1.7%, 95% CI: -3.54 to -0.18), p = 0.033, declining from 23.94 to 17.80 per 1,000 person-years. The analysis revealed that older age groups experienced more pronounced declines in statin initiation, while younger groups showed minimal changes (Fig 5).

### Sensitivity analysis of cumulative defined daily dose statin exposure

**Fig 6:**
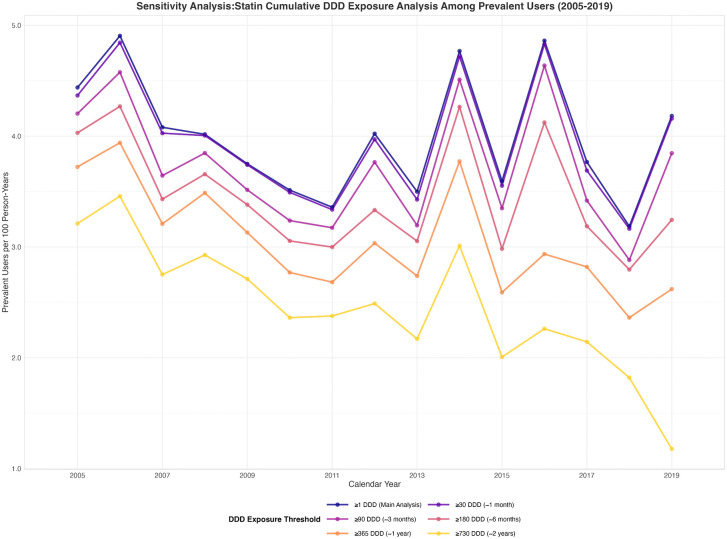
Statin cDDD exposure among prevalent users.

**Fig 7:**
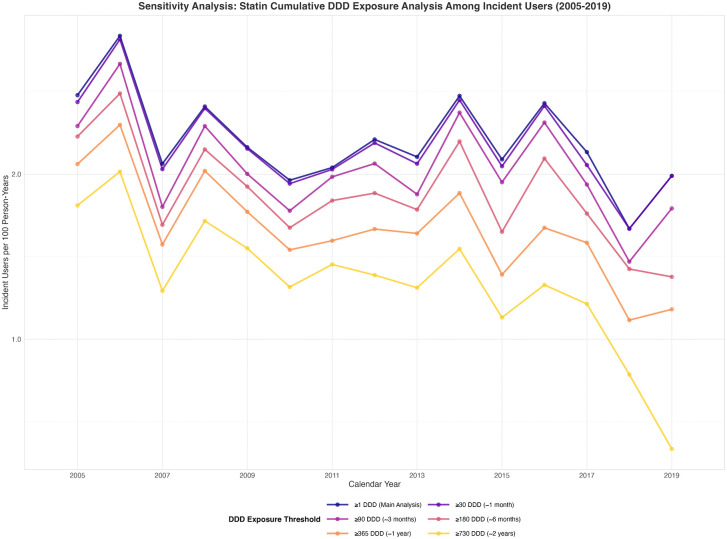
Statin cDDD exposure among incident users.

The parallel temporal patterns of all cDDD thresholds validated the robustness of observed statin prescription trends (Fig 6, Fig 7). The consistent proportional relationships between thresholds across the study period suggest that approximately 30–56% of statin initiations result in discontinuation before achieving one year of cumulative exposure (S5 Table, S6 Table).

#### Prevalent users.

We quantified the proportion of prevalent statin users among osteoporosis patients who achieving different cDDD thresholds (Fig 6). The proportion of users peaked in 2006 at 4.9 per 100-person years and declining to 4.2 per 100 person-years by 2019 in the main analysis (≥1 cDDD). Higher cDDD thresholds showed lower proportion of users over the study period. Significant decreasing trends were observed in subgroups with cDDD ≥ 365 (p < 0.001) and cDDD ≥ 730 (p < 0.001). The magnitude of decline was most pronounced for long-term users who with more than 2 year statin exposure, with ≥730 cDDD, showing a 33% reduction compared to 21% overall in the main analysis (Table 3).

**Table 3:** Temporal trends analysis on cumulative defined daily dose of statin exposure analysis using joinpoint regression.

	cDDD Threshold	Year	AAPC	(95% CI)	*p value*
**Prevalence** **users**	cDDD ≥ 1	2005–2019	−0.75	(−2.49, 1.01)	0.37
	cDDD ≥ 30	2005–2019	−0.75	(−2.48, 1.01)	0.37
	cDDD ≥ 90	2005–2019	−0.87	(−2.72, 1.02)	0.34
	cDDD ≥ 180	2005–2019	−1.29	(−2.96, 0.41)	0.12
	cDDD ≥ 365	2005–2019	−2.40*	(−3.81, −0.96)	<0.001
	cDDD ≥ 730	2005–2016	−4.76*	(−6.67, −2.82)	<0.001
**Incidence users**	cDDD ≥ 1	2005–2019	−1.46	(−2.88, −0.02)	0.05
	cDDD ≥ 30	2005–2019	−1.47	(−2.88, −0.04)	0.05
	cDDD ≥ 90	2005–2019	−1.58	(−3.28, 0.15)	0.07
	cDDD ≥ 180	2005–2019	−2.32*	(−3.97, −0.63)	0.01
	cDDD ≥ 365	2005–2019	−3.25*	(−4.86, −1.60)	<0.001
	cDDD ≥ 730	2005–2019	−6.73*	(−10.31, −3.01)	<0.001

#### Incident users.

Among incident users, we quantified the proportion of treatment-naive osteoporosis patients achieving different cDDD thresholds (Fig 7). The rate of patients initiating statins (≥1 cDDD) peaked in 2006 with 2.8 per 100 person-years at risk in the main analysis (≥1 cDDD and declined to 2.0 per 100 person-years by 2019. The proportion achieving higher cDDD thresholds significantly decreased over time for sustained exposure categories (cDDD ≥ 180, p = 0.01*;* cDDD ≥ 365, p < 0.001). However, the steepest declines occurred among patients who eventually achieved long-term exposure, with ≥730 cDDD declining by 90% compared to 20% for ≥1 cDDD at the end of study period (Table 3).

## Discussion

Our analysis leveraged a large sample size that provided robust statistical power to detect temporal trends. Importantly, statistical significance should be interpreted within the framework of clinical relevance. The observed annual percentage changes in statin prevalence (AAPC: -1.03%) and incidence (AAPC: -1.82%) present significant declining trends of statin prescription. The cumulative impact of these changes over the 15-year study period suggests a shift of prescribing practice rather than random variation. Notable patterns emerged across demographic subgroups, with the 70–79 age group experiencing the steepest declines despite initially highest rates, and male prescription rates declining more rapidly than female rates (male AAPC: -2.77% vs. female AAPC: -0.8%), leading to narrowing of the gender gap by 2019. The peak statin prescription observed in 2006 and 2014 coincided with NICE guideline changes of statin prescription in our osteoporosis cohort. The NICE guideline update in January 2006 represented a pivotal shift in UK statin prescription recommendations [20,33], directing general practitioners to prescribe statins to adults under 75 years whose 10-year cardiovascular disease risk exceeded 20%, as calculated using established risk prediction tools like the Framingham risk score [33]. A more dramatic transformation occurred in July 2014, when NICE substantially lowered this threshold to recommend statin therapy for adults with a 10-year CVD risk of just 10% or greater—effectively doubling the eligible population [34].

The cumulative defined daily dose (cDDD) sensitivity analysis revealed that statin initiation and maintenance patterns among osteoporosis patients remained consistent across different exposure thresholds with the temporal trends aligning with these UK guideline changes [34]. The steeper declines observed among patients achieving higher cDDD thresholds suggest a growing disparity between statin initiation and long-term continuation. While short-term treatment remained relatively stable, sustained treatment (≥365 days) decreased substantially over time. Despite these guideline expansions promoting broader statin use, the fundamental challenges of treatment persistence among osteoporosis patients remained unresolved [35]. The consistently high discontinuation rates across all time periods indicate significant gaps in cardiovascular protection for this high-risk population. These findings highlight systemic barriers to long-term adherence and the need for adherence-focused interventions rather than solely emphasizing increased prescribing rates.

Compared to O’Keeffe et al. study, the statin prescription rates in our osteoporosis cohort is substantially lower than those observed in aged-matched cohorts in the general UK population [19]. O’Keeffe et al. reported declining incidence rates from 2005 to 2013 with a peak in 2006 [19]. However, they observed a paradoxical increase in prevalence over the same period, a finding that remained unexplained in their analysis [19]. In contrast, our osteoporosis population demonstrated significant lower prescription rate with declining trends in both prevalence and incidence rates from 2005–2019.

The overall declining trends of incidence rate and prevalence reported in our analysis reflect a more restrictive implementation of statin prescribing guidelines in osteoporosis patients. Emerging evidence regarding statin-associated adverse effects, including potential impacts on bone metabolism and muscle-related symptoms, may have influenced prescribing patterns in osteoporosis patients who are already at increased risk for musculoskeletal complications. The frequent reports of statin-associated muscle symptom began to significantly influence prescribing decisions, particularly in osteoporosis patients already at elevated fracture risk [36–38]. Studies also reported that muscle-related side effects were experienced by 60% of former statin users and 25% of current users, with side effects constituting the primary reason for discontinuation (62%) among those who stopped therapy. The emergence of rare but severe adverse effects, including rhabdomyolysis and statin-induced necrotizing autoimmune myopathy, further heightened clinical vigilance, even though these conditions affect only a small minority of patients [39,40]. Throughout this period, emerging safety concerns regarding diabetes mellitus and central nervous system manifestations likely increased prescriber caution, particularly when initiating therapy in older adults [13,40,41].

Our study identified a gender-based prescribing patterns, with males demonstrating higher statin prescription rates than females. The findings of O’Keeffe et al. aligned with our observation of generally greater prescription rates among men [19]. Clinicians may have regarded male osteoporosis patients as presenting with an enhanced cardiovascular comorbidity profile, thus justifying more intensive lipid-lowering therapeutic interventions [24,42]. However, our study documented notable narrowing of gender gap in the latter years, with male prescription rates declining more rapidly than female rates by 2019 (male prevalence AAPC: -2.77% vs. female AAPC: -0.8%). The wider confidence observed in male subgroup estimate (prevalence: 95% CI:-4.64, -0.86; incidence: 95% CI:-5.90, -0.86) compared to female reflects the smaller number of males in the osteoporosis cohort, indicating reduced statistical precision for this subgroup. This convergence likely reflects several interconnected developments in clinical practice. The evolving understanding of shared bone-cardiovascular pathophysiology and implementation of more systematic, evidence-based protocols. Specifically, traditional risk assessment tools like the Framingham Risk Score, which were developed predominantly using male cohorts, systematically underestimated cardiovascular risk in postmenopausal women with osteoporosis [43,44]. Concurrently, growing evidence demonstrating that osteoporosis serves as an independent predictor of cardiovascular events, particularly in postmenopausal women, has heightened clinical awareness [45,46]. These advances have collectively contributed to more equitable treatment approaches across genders.

The study period coincided with major guideline updates, including the 2013 ACC/AHA Cholesterol Guidelines, which emphasized comprehensive risk assessment algorithms that better account for female-specific risk factors and recognized elevated cardiovascular risk in women with early menopause, leading to more equitable treatment approaches across genders [23,24,45–47]. The study period also coincided with the 2014 NICE guidelines, which introduced more nuanced recommendations emphasizing individualized risk-benefit assessment [34]. These evolving guidelines had differential impact across demographic groups. The steeper decline in male statin prescription rates (prevalence AAPC: -2.77%; incidence AAPC: -3.41%) likely reflects correction of historical overprescribing due to systematic cardiovascular risk overestimation in males [48,49]. The updated criteria may have disproportionately influenced clinical decision-making for male osteoporosis patients, with the observed gender convergence representing clinical recalibration toward more appropriate, individualized prescribing practices.

Age-stratified analysis revealed divergent temporal patterns across age groups. Younger osteoporosis patients showed relatively stable statin prescription trends without significant decline (50–59 years: AAPC -0.92% for prevalence, -0.41% for incidence; 60–69 years: AAPC -0.32% for prevalence, -1.13% for incidence). The youngest age group (50–59 years) exhibited the widest confidence intervals (prevalence 95% CI: -2.90, 1.10; incidence 95% CI: -2.52, 1.75). The insignificant findings of this age group may therefore indicate reduced statistical precision due to smaller sample size rather than absence of true temporal trends. Notably, however, a breakpoint in 2018 specially observed in this age group, likely reflecting that the implementation of the 2018 ACC/AHA cholesterol guidelines may alter prescribing behaviour for osteoporosis patients. This guideline changed statin prescribing by mandating 10-year ASCVD risk stratification using the Pooled Cohort Equations, with a ≥ 7.5% threshold for treatment initiation in primary prevention [12,50]. However, osteoporosis was not included as a cardiovascular risk factor despite epidemiological evidence showed that osteoporosis patients with elevated cardiovascular risk [5,47,51]. This exclusion creates systematic risk underestimation, particularly problematic for younger patients (50–59 years) who accumulate lower age-related risk score [50]. For example, a woman with osteoporosis diagnosed at 59 years old, but otherwise low cardiovascular profile may be estimated with a 10-year ASCVD risk of <5%, which is below statin initiation threshold despite her chronic condition signalling elevated cardiovascular risk.

While the 70–79 years age group exhibited the highest initial prevalence among all age strata, consistent with findings from O’Keeffe et al.[19], our analysis revealed significant declines in statin prescription in older osteoporosis cohorts (70–79: AAPC -2.77% for incidence, -1.66% for prevalence; 80 + : AAPC -1.7% for incidence, -0.96% for prevalence), which may reflect multiple converging clinical considerations that emerged during the study period. The growing emphasis on deprescribing initiatives in geriatric medicine contributed to increased scrutiny of statin therapy in older patients with multiple comorbidities, where polypharmacy burden often outweighs potential therapeutic benefits. Patients with multimorbidity represent a complex clinical scenario, especially among older osteoporosis patients who typically receive 4–6 concurrent medications for chronic diseases such as hypertension, diabetes, chronic kidney disease, and arthritis [52].

Moreover, O’Keeffe et al. revealed that the substantially lower statin prescription rates in osteoporosis patients compared to age-matched general population, who are individuals with similar age but without osteoporosis [19]. This indicated that multiple guideline expansions during the study period did not translate into increased cardiovascular protection for this vulnerable population, and highlighted a potential therapeutic gap which clinicians should recognize when managing cardiovascular risk in osteoporosis patients. The alignment between prescription peaks and guidelines changes demonstrated healthcare system responsiveness to evolving evidence. However, the differential patterns across age groups highlight the need for age-specific cardiovascular risk stratification tools in osteoporosis management. Policy makers should prioritize developing integrated care pathways that harmonize osteoporosis and cardiovascular risk management, moving beyond single-disease approaches toward comprehensive risk assessment frameworks.

While our findings document significant declines in statin prescribing among osteoporosis patients, it is critical to appraise whether these trends represent appropriate risk-based prescribing or a missed therapeutic opportunity. This evaluation is particularly important given emerging evidence that statins may offer dual benefits in osteoporosis patients beyond their established cardiovascular effects. The pleiotropic effects of statins significantly affected bone turnover and regeneration processes, impacting crucial cell types such as mesenchymal stem cells, osteoblasts, endothelial cells, and osteoclasts [53]. Additionally, statins have shown potential anti-inflammatory and antimicrobial properties, which could be beneficial in treating infections that disrupt normal bone healing [54]. Furthermore, meta-analysis indicates that the combination of statin and anti-osteoporotic treatment increased bone mineral density, and offered a safer treatment profile for osteoporosis patients with multiple chronic conditions [55–57]. The declining prescription trends of statin documented in our study raise concerns about potential underutilization of a medication with dual protection in the osteoporosis population.

## Clinical and policy implications

This study provides valuable descriptive epidemiology of statin prescribing patterns in osteoporosis patients through comprehensive analysis of the IMRD database, revealing important temporal trends in both incidence and prevalence. The substantially lower statin prescription rates in osteoporosis patients compared to the age-matched general population highlight a potential therapeutic gap, suggesting that clinicians may be missing opportunities to manage both cardiovascular risk and bone health in this population. The alignment between prescription peaks and guidelines changes demonstrated healthcare system responsiveness to evolving evidence. However, the differential patterns across age groups highlight the need for age-specific cardiovascular risk stratification tools in osteoporosis management. Policy makers should prioritize developing integrated care pathways that harmonize osteoporosis and cardiovascular risk management. The integrated care pathway should consider embedding routine cardiovascular risk assessment in the osteoporosis diagnosis and management protocols with mandatory screening at the time of osteoporosis diagnosis. Cardiovascular risk calculators, such as the Pooled Cohort Equations, should be updated to incorporate osteoporosis as an independent risk factor, acknowledging its association with elevated cardiovascular risk beyond traditional risk factors. Clinical decision supporting system should alert physicians the potential skeletal benefits of statin when prescribing statin for cardiovascular indication. These structural changes advance chronic disease management from single-disease approach toward integrated care in osteoporosis population.

## Limitations

This study benefits from utilizing IMRD database, which has been demonstrated in multiple large-scale studies to provide robust data for prescribing trends and has been validated against national statistics [29,58–60]. This substantial sample size enables robust stratification across key variables including age and gender. However, several methodological limitations warrant consideration when interpreting our findings. Our analyses relied on Read codes recorded by general practitioners, which introduces potential variability stemming from differences in coding behaviours between individual clinicians and practices. Our study period cut-off was in 2019, the year before the transition of UK primary care medical recording system from Read Codes to SNOMED CT, with full implementation completed by April 2020. As our analysis excluded prescriptions recorded with SNOMED CT codes, this may have introduced modest underestimation of statin prescribing in the latter part of the study period. Furthermore, our methodology captures only prescriptions issued within primary care settings, potentially missing prescriptions initiated in secondary care but not subsequently recorded in GP systems. Several unmeasured factors that may influence statin prescribing behaviour were not addressed in our analysis, including socioeconomic status, comprehensive comorbidity burden, and polypharmacy. These factors can affect medication access, clinical contraindications, and treatment prioritization. Future studies should incorporate these variables to better distinguish evidence-based clinical decision-making from access barriers or missed therapeutic opportunities.

## Conclusions

This study examined the IMRD data of UK spanning 15 years from 2005 to 2019, revealing declining trend of statin use with high discontinuation rate among osteoporosis patients suggests that multiple guideline expansions during the study period did not translate into increased cardiovascular protection for this vulnerable population. Our analysis showed a notable gender convergence and age-specific variations over the study period, while the patients aged 70–79 years had the highest initial prescription rates but experienced the steepest subsequent declines. As healthcare system is tackling the challenge from the aging populations with multimorbidity, this study underscores the importance of moving beyond single-disease focused approaches toward more nuanced strategies that account for the clinical realities of treating patients with overlapping conditions.

## Supporting information

S1 Text Read codes of osteoporosis. (DOCX)

S2 Text Read codes of statins. (DOCX)

S1 Table Statin prescription prevalence and incidence rate. (XLSX)

S2 Table Statin prescription prevalence and incidence rate stratified by gender. (XLSX)

S3 Table Statin prescription prevalence stratified by age groups. (XLSX)

S4 Table Statin prescription incidence rate stratified by age groups. (XLSX)

S5 Table Statin cDDD exposure among prevalent users. (XLSX)

S6 Table Statin cDDD exposure among incident users. (XLSX)
