# Supplementary material for: Trends in statin prescription among osteoporosis patients: A retrospective cohort study using UK primary care data
Source: PLOS Glob Public Health. 2025 Dec 29;5(12):e0005656. doi: 10.1371/journal.pgph.0005656 (PMC12747384; doi:10.1371/journal.pgph.0005656)
Supplement: S1 Text — (DOCX) [file pgph.0005656.s001.docx]

S1 Text. Read codes of osteoporosis.

**Read code description**

585O.00 Quantitative ultrasound scan of heel - result osteoporotic

58E4.00 Forearm DXA scan result osteoporotic

58EA.00 Heel DXA scan result osteoporotic

58EG.00 Hip DXA scan result osteoporotic

58EM.00 Lumbar DXA scan result osteoporotic

58EV.00 Femoral neck DEXA scan result osteoporotic

66a2.00 Osteoporosis treatment started

66a3.00 Osteoporosis treatment stopped

66a4.00 Osteoporosis treatment changed

66a5.00 Osteoporosis - no treatment

66a6.00 Osteoporosis - dietary advice

66a7.00 Osteoporosis - dietary assessment

66a8.00 Osteoporosis - exercise advice

66a9.00 Osteoporosis - falls prevention

66aA.00 Osteoporosis - treatment response

66aB.00 Osteoporosis - no treatment response

9kj0.00 Bone sparing drug treatment offered for osteoporosis - ESA

N330.00 Osteoporosis

N330000 Osteoporosis, unspecified

N330100 Senile osteoporosis

N330200 Postmenopausal osteoporosis

N330300 Idiopathic osteoporosis

N330400 Dissuse osteoporosis

N330500 Drug-induced osteoporosis

N330600 Postoophorectomy osteoporosis

N330700 Postsurgical malabsorption osteoporosis

N330800 Localized osteoporosis - Lequesne

N330900 Osteoporosis in multiple myelomatosis

N330A00 Osteoporosis in endocrine disorders

N330B00 Vertebral osteoporosis

N330C00 Osteoporosis localized to spine

N330D00 Osteoporosis due to corticosteroids

N330z00 Osteoporosis NOS

N331200 Postoophorectomy osteoporosis with pathological fracture

N331300 Osteoporosis of disuse with pathological fracture

N331400 Postsurgical malabsorption osteoporosis with path fracture

N331500 Drug-induced osteoporosis with pathological fracture

N331600 Idiopathic osteoporosis with pathological fracture

N331800 Osteoporosis + pathological fracture lumbar vertebrae

N331900 Osteoporosis + pathological fracture thoracic vertebrae

N331A00 Osteoporosis + pathological fracture cervical vertebrae

N331B00 Postmenopausal osteoporosis with pathological fracture

N331M00 Fragility fracture due to unspecified osteoporosis

N331M11 Minimal trauma fracture due to unspecified osteoporosis

N374600 Osteoporotic kyphosis

NyuB000 [X]Other osteoporosis with pathological fracture

NyuB100 [X]Other osteoporosis

NyuB200 [X]Osteoporosis in other disorders classified elsewhere

NyuB800 [X]Unspecified osteoporosis with pathological fracture
