# Supplementary material for: Trends in statin prescription among osteoporosis patients: A retrospective cohort study using UK primary care data
Source: PLOS Glob Public Health. 2025 Dec 29;5(12):e0005656. doi: 10.1371/journal.pgph.0005656 (PMC12747384; doi:10.1371/journal.pgph.0005656)
Supplement: S2 Text — (DOCX) [file pgph.0005656.s002.docx]

S2 Text. Read codes of statins.

**Read code Description**

20801978 Rosuvastatin 5mg tablets

20822978 Rosuvastatin 10mg tablets

29373978 Atorvastatin 20mg tablets

34062978 Fenofibrate 145mg / simvastatin 40mg tablets

34063978 Fenofibrate 145mg / simvastatin 40mg tablets

34065978 Fenofibrate 145mg / simvastatin 20mg tablets

52002978 Simvastatin 40mg tablets

52003978 Simvastatin 20mg tablets

56193979 Atorvastatin 80mg tablets

57832979 Atorvastatin 20mg tablets

57833979 Atorvastatin 10mg tablets

57838979 Atorvastatin 40mg tablets

57851979 Atorvastatin 80mg tablets

58088979 Atorvastatin 10mg tablets

58151979 Atorvastatin 60mg tablets

58153979 Atorvastatin 30mg tablets

58650979 Atorvastatin 20mg tablets

58651979 Atorvastatin 20mg tablets

58653979 Atorvastatin 20mg tablets

58654979 Atorvastatin 20mg tablets

58655979 Atorvastatin 20mg tablets

58656979 Atorvastatin 20mg tablets

58658979 Atorvastatin 10mg tablets

58660979 Atorvastatin 10mg tablets

58661979 Atorvastatin 10mg tablets

58662979 Atorvastatin 10mg tablets

58668979 Atorvastatin 40mg tablets

58669979 Atorvastatin 40mg tablets

58671979 Atorvastatin 40mg tablets

58672979 Atorvastatin 40mg tablets

58673979 Atorvastatin 40mg tablets

58706979 Atorvastatin 80mg tablets

58709979 Atorvastatin 80mg tablets

58710979 Atorvastatin 80mg tablets

58711979 Atorvastatin 80mg tablets

59480979 Fluvastatin 20mg capsules

61212979 Atorvastatin 20mg chewable tablets sugar free

61213979 Atorvastatin 20mg chewable tablets sugar free

61215979 Atorvastatin 10mg chewable tablets sugar free

61484979 Pravastatin 40mg tablets

61489979 Simvastatin 20mg tablets

61490979 Simvastatin 10mg tablets

62570979 Simvastatin 40mg/5ml oral suspension sugar free

62597979 Simvastatin 20mg/5ml oral suspension sugar free

63597979 Pravastatin 40mg/5ml oral suspension

64582979 Atorvastatin 40mg/5ml oral suspension

64584979 Atorvastatin 40mg/5ml oral solution

64586979 Atorvastatin 10mg/5ml oral suspension

64588979 Atorvastatin 10mg/5ml oral solution

64634979 Atorvastatin 20mg/5ml oral suspension

64636979 Atorvastatin 20mg/5ml oral solution

64839979 Simvastatin 40mg/5ml oral suspension sugar free

79254979 Simvastatin 20mg/5ml oral suspension sugar free

79256979 Simvastatin 10mg/5ml oral suspension

81048998 Atorvastatin 20mg chewable tablets sugar free

81049998 Atorvastatin 20mg chewable tablets sugar free

81050998 Atorvastatin 10mg chewable tablets sugar free

81051998 Atorvastatin 10mg chewable tablets sugar free

82592978 Atorvastatin 20mg tablets

82593978 Atorvastatin 10mg tablets

82907998 Fluvastatin 80mg modified-release tablets

83030998 Simvastatin 80mg tablets

83099998 Simvastatin 40mg/5ml oral suspension sugar free

86020998 Simvastatin 20mg/5ml oral solution sugar free

86467998 Rosuvastatin 5mg tablets

86468998 Rosuvastatin 5mg tablets

86787998 Simvastatin 80mg / Ezetimibe 10mg tablets

86788998 Simvastatin 40mg / Ezetimibe 10mg tablets

86789998 Simvastatin 20mg / Ezetimibe 10mg tablets

86791998 Simvastatin 80mg / Ezetimibe 10mg tablets

86794998 Simvastatin 80mg / Ezetimibe 10mg tablets

86795998 Simvastatin 40mg / Ezetimibe 10mg tablets

86796998 Simvastatin 40mg / Ezetimibe 10mg tablets

86797998 Simvastatin 20mg / Ezetimibe 10mg tablets

86798998 Simvastatin 20mg / Ezetimibe 10mg tablets

87373998 Simvastatin 10mg tablets

87417998 Simvastatin 20mg tablets

87418998 Simvastatin 10mg tablets

87916998 Simvastatin 40mg tablets

87917998 Simvastatin 20mg tablets

87918998 Simvastatin 10mg tablets

88534998 Rosuvastatin 10mg tablets

89119979 Simvastatin 20mg / Ezetimibe 10mg tablets

89306996 Atorvastatin 40mg tablets

89306997 Atorvastatin 20mg tablets

89306998 Atorvastatin 10mg tablets

89311996 Atorvastatin 40mg tablets

89311997 Atorvastatin 20mg tablets

89311998 Atorvastatin 10mg tablets

89321979 Rosuvastatin 20mg tablets

90309998 Atorvastatin 80mg tablets

90310998 Atorvastatin 80mg tablets

90973998 Rosuvastatin 20mg tablets

91194998 Fluvastatin 80mg modified-release tablets

92154990 Simvastatin 20mg/5ml oral suspension sugar free

92220998 Simvastatin 80mg tablets

92408998 Rosuvastatin 20mg tablets

92409998 Rosuvastatin 10mg tablets

92410998 Rosuvastatin 40mg tablets

92471998 Simvastatin 80mg tablets

92539998 Rosuvastatin 40mg tablets

92804996 Fluvastatin 80mg modified-release tablets

92804997 Fluvastatin 40mg capsules

92804998 Fluvastatin 20mg capsules

92805997 Fluvastatin 40mg capsules

92805998 Fluvastatin 20mg capsules

93243996 Pravastatin 40mg tablets

93243997 Pravastatin 20mg tablets

93243998 Pravastatin 10mg tablets

93244996 Pravastatin 40mg tablets

93244997 Pravastatin 20mg tablets

93244998 Pravastatin 10mg tablets

93619996 Simvastatin 40mg tablets

93619997 Simvastatin 20mg tablets

93619998 Simvastatin 10mg tablets

93620996 Simvastatin 40mg tablets

93620997 Simvastatin 20mg tablets

93620998 Simvastatin 10mg tablets

93871990 Simvastatin 40mg tablets

93873990 Simvastatin 10mg tablets

94407990 Simvastatin 20mg tablets

94782990 Pravastatin 20mg tablets

94789990 Pravastatin 10mg tablets

94830990 Pravastatin 20mg tablets

94831990 Pravastatin 10mg tablets

94849990 Pravastatin 40mg tablets

94850990 Pravastatin 20mg tablets

94851990 Pravastatin 10mg tablets

94920990 Simvastatin 20mg tablets

94927990 Simvastatin 80mg tablets

95185990 Simvastatin 80mg tablets

97382979 Atorvastatin 20mg tablets

97399979 Atorvastatin 10mg tablets

97403979 Fluvastatin 80mg modified-release tablets

97424979 Fluvastatin 20mg capsules

97430979 Fluvastatin 20mg capsules

97454979 Pravastatin 20mg tablets

97455979 Pravastatin 10mg tablets

97476979 Simvastatin 40mg tablets

97478979 Simvastatin 40mg tablets

97482979 Simvastatin 40mg tablets

97487979 Simvastatin 40mg tablets

97508979 Simvastatin 10mg tablets

97513979 Simvastatin 10mg tablets

97514979 Simvastatin 10mg tablets

97518979 Simvastatin 10mg tablets
